# Self-Reported General Health, Overall and Work-Related Stress, Loneliness, and Sleeping Problems in 335,625 Swedish Adults from 2000 to 2016

**DOI:** 10.3390/ijerph17020511

**Published:** 2020-01-14

**Authors:** Victoria Blom, Lena V. Kallings, Björn Ekblom, Peter Wallin, Gunnar Andersson, Erik Hemmingsson, Örjan Ekblom, Jonas Söderling, Elin Ekblom Bak

**Affiliations:** 1 Åstrand Laboratory of Work Physiology, The Swedish School of Sport and Health Sciences, 114 33 Stockholm, Sweden; lena.kallings@gih.se (L.V.K.); bjorn.ekblom@gih.se (B.E.); erik.hemmingsson@gih.se (E.H.); orjan.ekblom@gih.se (Ö.E.); Elin.EkblomBak@gih.se (E.E.B.); 2 Division of Insurance medicine, Department of Clinical Neuroscience, Karolinska Institutet, 171 77 Stockholm, Sweden; 3 The Research Dept, HPI Health Profile Institute AB, 182 53 Stockholm, Sweden; peter.wallin@hpihealth.se (P.W.); gunnar.andersson@hpihealth.se (G.A.); 4 Department of Medicine, Karolinska institutet, Karolinska University Hospital, Solna, 171 77 Stockholm, Sweden; soderling.jonas@gmail.com

**Keywords:** public health, self-reported health, sleeping problems, stress, loneliness, working population

## Abstract

The prevalence of poor health, in particular stress-related mental ill-health, is increasing over time and birth cohorts. As rapid societal changes have occurred in the last decade and still are occurring, there is an interest in investigating the trends in health-related factors. The aim of the present study was to investigate trends in self-reported general health, overall stress, work-related stress, feelings of loneliness, and sleeping problems in 335,625 Swedish adults across categories of gender, geographic regions, length of education, and age from 2000 to 2016. On population level, sleeping problems and poor general health have increased markedly and significantly, while experiences of work stress decreased between 2000 and 2016 (*p* < 0.05). Overall stress and level of loneliness were unchanged (*p* > 0.05). The risk of having ≥3 symptoms (any of poor or very poor general health, often or very often perceived overall stress, loneliness, or sleeping problems) has increased significantly from 2000 to 2016 (ß = 1034 (1027–1040)). This increase was significantly higher in young (ß = 1052 (1038–1065)) and individuals with lower education (ß = 1056 (1037–1076)) compared to older and high length of education.

## 1. Introduction

The prevalence of poor health, in particular stress-related mental ill-health, is increasing over time and birth cohorts. As rapid societal changes have occurred in the last decade and are still occurring, there is an interest in investigating the trends in health-related factors. Particularly, increases in solitary living, growing urbanization, and social media suggest changing trends in general health, stress, feelings of loneliness, and sleeping problems [1]. Few studies have taken a broad picture describing various health factors together including demographics, which may allow further understanding of the trends.

Perceived general health, defined as overall physical and mental health, is associated with multiple health outcomes, such as cardiovascular disease, health-related quality of life, and all-cause mortality. Perceived general health is deteriorating in many countries, particularly in lower educational and manual work groups [1]. Moreover, stress related disorders are today the most common reasons for long-term sick leave across the European Region with a sharp increase in Sweden in the last ten years [2,3,4,5,6]. In particular, women and middle aged individuals (30–49) represent a larger amount of the sickness absence due to stress-related ill-health as well as report more stress reactions [6,7]. However, self-reported stress reactions have increased in particular among the youngest [8,9].

Perceived loneliness, defined as living without companionship, social support, or social connectedness [10], has shown to be a stressor and a strong predictor of poor general health, long-term morbidity, and mortality in the general population, comparable to established risk factors such as hypertension and excessive alcohol consumption [11,12,13,14]. Across Finnish surveys, the prevalence in loneliness increased from 4.4% in 1991 to 7.2% in 2014 [12].

A common stress reaction is poor sleep quality, which can be defined as time to onset of sleep, number of awakenings, and length of sleep, associated with clinical and subclinical mood status, anxiety disorders, and behavioral problems and predispose to psychiatric disorders later in life [15]. Sleeping problems are widely believed to impair health through various effects on, e.g., neuroendocrine, immune, and metabolic systems [16] and is related to sickness absenteeism [17]. A study in an adult population in Norway showed significant increases in insomnia over a 10-year study period; the prevalence of sleep-onset insomnia increased from 13.1% to 15.2%, dissatisfaction with sleep from 8.2% to 13.6%, and daytime impairment from 14.8% to 18.8% [18]. Similarly, a Swedish study showed a doubling of the prevalence in women reporting sleeping problems from 1968 to 2004 [19] and in study in USA, encounters for insomnia increased between 2005 to 2014, in particular in women and individuals over 40 years [20].

The aim of the present study was to investigate trends in self-reported general health, overall stress, work-related stress, feelings of loneliness, and sleeping problems in 335,625 Swedish adults across gender affiliation, regions, lengths of education, and age groups during 2000–2016. Based on previous studies, the hypotheses are that all health factors show a negative trend of poorer general health, more overall and work-related stress, more feelings of loneliness, and sleeping problems. Individuals with lower education, women, and young individuals are hypothesized to experience greater reductions in subjective health ratings than other education groups, men, and older individuals. The present study thus adds to the literature by taking a broad picture in a large sample describing various health factors together including demographics, allowing further understanding how these co-occur over time.

## 2. Method

### 2.1. Material

This study was based on data from Health Profile Assessments (HPAs) carried out in Swedish health services since the middle of the 1970s [21]. The HPA is an interdisciplinary method and includes an extensive questionnaire, measurements of anthropometrics and blood pressure, a submaximal cycle test for estimation of VO_2_max, and a person-centered dialogue with an HPA coach. Participation in the HPAs was offered to all employees working for a company or an organization connected to occupational or other health service in Sweden, and free of charge for the employee. All data were subsequently recorded in the Health Profile Institute database. From October 1982 to December 2016, 503,432 participants (18–74 years old) with a first-time HPA and providing data on gender, age, educational level, and region were stored in the central database. The annual rate of participants was substantially lower in the first years, 1982 (*n* = 1) and 1999 (*n* = 5247), compared to the following full years, 2000 (*n* = 7640) and 2016 (*n* = 34 186). To minimize influence of uncertainty and variations in the data collection procedure, we limited our primary analyses to 2000–2016 (*n* = 488 372). Table S2 presents data for 1995–1999. Of these, 69% (*n* = 335 625) provided valid data of the health variables and were included in the analyses. Drop out analyses have been carried out in the data and described elsewhere [22]. All participants provided informed consent prior to data collection. The study was approved by the ethics board at the Stockholm Ethics Review Board (Dnr 2015/1864-31/2 and 2016/9-32), and adhered to the Declaration of Helsinki.

### 2.2. Main Outcomes

The main outcomes in the present trend study was derived from the questionnaire, including the health variables Sleeping problems stated as “I have sleeping problems”, Overall stress stated as “I experience stress in my life in total, work included”, Work-related stress stated as “I experience stress at work”, and Feelings of loneliness stated as “I experience feelings of loneliness in my life in total, work included”. These were answered on a five-point Likert scale ranging from Very often to Never. These were dichotomized as 1–2 including often or very often and 3–5 including sometimes, rarely, and never. Moreover, one question of general health was stated as “I experience that my health concerning body and soul is…”and answered on a five-point Likert scale ranging from very bad to very good. These were dichotomized as 1–2 including poor or very poor general health and 3–5 including neither good nor bad, good, or very good general health. The measures of health were self-reported and not psychometrically investigated. However, the HPA measures are scientifically published [21,22] and evaluated within the Health Profile Institute database since 1976. See Table 1 for sample description.

### 2.3. Other Measurements

Highest educational attainment and place of dwelling (as county in Sweden of residence) at the time for the HPA was obtained by linking the personal identity number of the participants with data from Statistics Sweden.

### 2.4. Statistical Analyses

For analyses of trends in health factors between 2000 and 2016, years were grouped into two-year periods for reducing variations between years and for increasing statistical power. Mean values of each health variable per two-year period were standardized, using the direct method, to the population 18–74 years old in Sweden in 2015 by sex, age (18–24 years, 25–34 years, 35–44 years, 45–49 years, 50–54 years, 55–64 years, and 65–74 years), and length of education (<9 years, 10–12 years, and ≥12 years). Standardized mean values were calculated to account for yearly variations in important prognostic variables (sex, age, education, and region). Standardized mean values were stratified by sex, age (18–34 years, 35–49 years, and 50–74 years), length of education (<9 years, 10–12 years, and ≥12 years), and county (counties categorized as including the three largest cities of Sweden “Urban,” counties including a majority of rural municipalities defined by Swedish Association of Local Authorities and Regions “Rural,” and all other counties “All other”). Logistic regression models were applied to study trends in poor general health as well as overall stress, stress at work, often perceived loneliness, and sleeping problems over the study period within the total population and across subgroups. The dichotomized outcome variables were introduced as dependent variable, and sex, age, educational level, region, and year performed as independent variables. Significant difference was defined as *p* < 0.05 for the performed year variable. To study the interaction between subgroups in differences of the dichotomized outcome variables, the procedure described by Altman and Bland was used introducing the OR for the performed year variable for each sub-group [23]. Significant interactions were defined as *p* < 0.05 for the interaction term, and *p* < 0.017 for multiple comparisons. To study the trends in health variables per year between different subgroups, the probability values were computed for the difference between the B-coefficients. The statistical analyses were conducted using IBM SPSS (Statistical Package for the Social Sciences for Windows), version 24.0.0, 2016, SPSS Inc, Chicago, IL and SAS version 9.4.

## 3. Results

The distribution across age groups, sex and socioeconomic groups is shown in Table 1.

**Figure 2:**
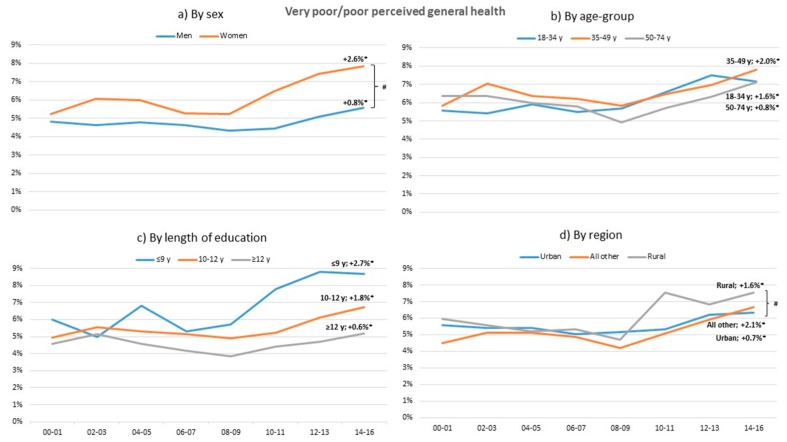
Change in standardized proportions of poor or very poor perceived general health between 2000–2001 and 2014–2016 in relation to sex, age group, length of education, and region. * Significant trend between 2000–2001 and 2014–2016 within sub-group (*p* < 0.05) # Significant trend difference between sub-groups, adjusted for multiple comparisons. All analyses are adjusted for the other sub-group variables.

During the study period, poor general health increased significantly in both women and men, all age groups, different length of education, and regions, with all trends mostly during the last eight years (Figure 2). Poor general health increased more in women than in men (*p* < 0.05). During 2014–2016, women experienced significantly higher level of poor general health than men.

**Figure 3:**
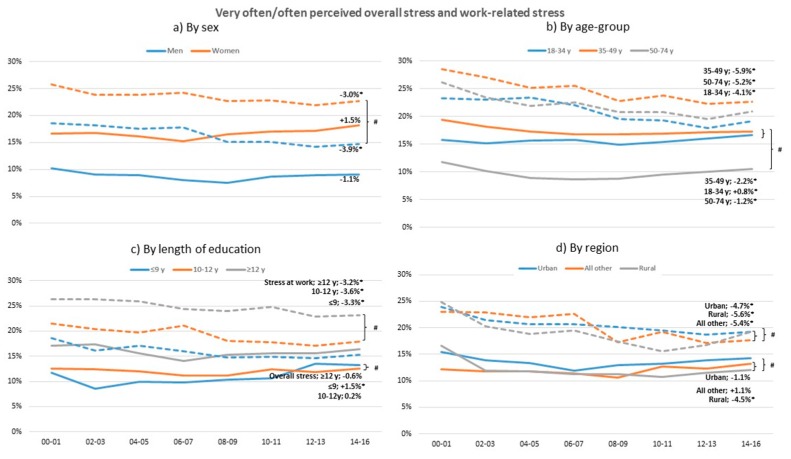
Change in standardized proportions of often or very often perceived overall stress (solid lines) and at work (dashed lines) between 2000–2001 and 2014–2016 in relation to sex, age group, length of education, and region. * Significant trend between 2000–2001 and 2014–2016 within sub-group, *p* > 0.05. # Significant trend difference between sub-groups, adjusted for multiple comparisons. All analyses are adjusted for the other sub-group variables.

Work-related stress decreased significantly in both sexes, all age groups, and all education groups between 2000 and 2016 (Figure 3). In 2000, over 25% of the women experience poor or very poor level of work stress but this decreased significantly by 3% in 2016 (*p* < 0.05). Men reported a decrease in work stress of 3.9%, which is significantly more than women (*p* < 0.02). The differences in overall stress is much less than work related stress or did not change at all. In individuals with low education as well as in the youngest age group, overall stress increased significantly over the years, for individuals with low education more than other education groups (*p* < 0.02), while the oldest age group and the rural region decreased in overall stress (*p* < 0.05). The oldest age group has the lowest level of overall stress compared to the other age groups (*p* < 0.05). Additional analyses showed that between 1995 and 2000 the stress prevalence was higher.

**Figure 4:**
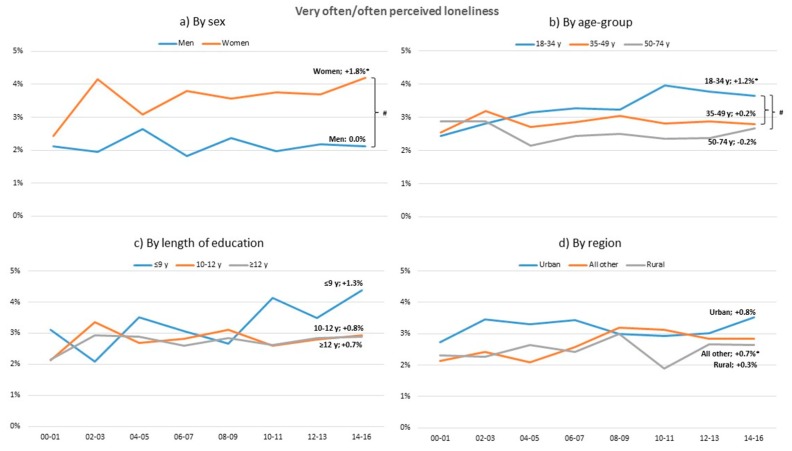
Change in standardized proportions of often or very often perceived loneliness between 2000–2001 and 2014–2016 in relation to sex, age-group, length of education and region. * Significant trend between 2000–2001 and 2014–2016 within sub-group (*p* < 0.05). # Significant trend difference between sub-groups, adjusted for multiple comparisons. All analyses are adjusted for the other sub-group variables.

Over the studied period perceived loneliness has increased in women, in the young age group and All other region. A higher level of loneliness is experienced in women than in men and in the young rather than the other age groups (Figure 4).

**Figure 5:**
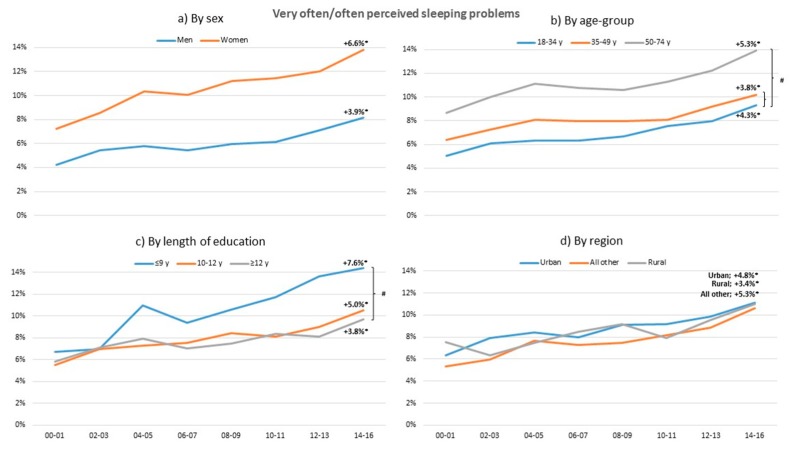
Change in standardized proportions of often or very often sleeping problems between 2000–2001 and 2014–2016 in relation to sex, age group, length of education, and region. * Significant trend between 2000–2001 and 2014–2016 within sub-group (*p* > 0.05). # Significant trend difference between sub-groups, adjusted for multiple comparisons. All analyses are adjusted for the other sub-group variables.

Over the years sleeping problems have increased in both genders, and all age and education groups and regions (Figure 5). The lowest education groups have the highest level of sleeping problems compared to other education groups and oldest age group has higher levels of sleeping problems compared to the other age groups.

**Figure 6:**
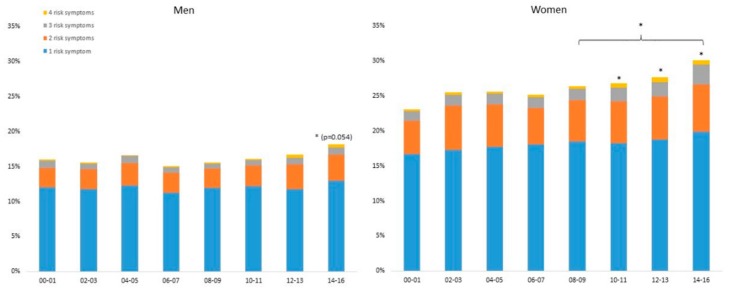
Standardized proportions of men (left) and women (right) with 1, 2, 3 and 4 risk symptoms, 2000–2016. A risk symptom is defined as poor or very poor perceived global health, often or very often perceived loneliness, overall stress or sleeping problems. * Significantly different from 2000–2001, or from other 2-year period if indicated with a bracket, *p* < 0.05.

Over the years, the number of risk symptoms (poor or very poor general health, often or very often perceived work and overall stress, loneliness, and sleeping problems) were higher in women than in men (Figure 6). Compared to 2000–2001, an increase in number of risk symptoms were seen in women during 2010–2011 and later, and for men during 2014–2016. For women, the number has also increased significantly from 2008–2009 to 2014–2016. 

In the population, the risk of having ≥3 symptoms (any of poor or very poor general health, often or very often perceived overall stress, loneliness, or sleeping problems) increased significantly from 2000 to 2016 (ß = 1034 (1027–1040) (see Table 2). This increase is significantly higher in the young and in those with low education compared to older and highly educated individuals.

Comparing OR for having no symptoms and three or more symptoms in the groups during the first four (2000–2003) and last four (2013–2016) years of the study period shows that men were more likely to have no symptoms and less likely to have three symptoms, compared to women, both in the first year and in the last year of the study period. Middle-aged participants were worse off in both the first and last years compared to the other age groups. While participants with different lengths of education had similar OR for no and three symptoms during 2000–2003, during 2013–2016, those with less education were significantly worse off (Table S1).

## 4. Discussion

The present study investigated different trends in self-reported general health, work related and overall stress, feelings of loneliness, and sleeping problems in Swedish adults across gender affiliation, regions, socioeconomic status groups, and age groups during 2000–2016. The results show that, at the population level, sleeping problems and poor general health increased markedly while experiences of work stress and loneliness decreased. These changes somewhat contradict each other, as sleeping problems, being a common stress reaction, and loneliness, being a common stressor, do not show the same trend as experiences of stress. One explanation for this could be that experiences of stress have become normalized, in particular among younger cohorts, which could impact the self-report of stress. The risk of having ≥3 symptoms (any of poor or very poor general health, often or very often perceived overall stress, loneliness, or sleeping problems) increased significantly more in individuals with low education and in young individuals between 18 and 34 years old compared to higher educated individuals and those over 35 years during 2000–2016. Women as compared to men had a higher level of loneliness and poor general health, with a more pronounced increase. Younger individuals reported a higher level than other age groups in feelings of loneliness and sleeping problems. Individuals with low education often experienced loneliness and sleeping problems compared to higher education. No clear differences between different regions were found, except that rural regions report poorer general health and urban regions report more feelings of loneliness than other regions and all other regions showed an increase in loneliness over the years. All regions showed increased levels of poor general health and sleeping problems. The hypotheses are thus partly confirmed. A negative trend in all health factors except loneliness and stress was found in the population. Individuals with lower education, women, and young individuals experienced greater reductions in subjective health ratings than other education groups, men, and older individuals.

### 4.1. Poor General Health

Poor general health is an independent predictor of mortality in numerous studies and poor health is more prevalent in low socioeconomic groups [24]. In the present study, in line with the literature [25,26,27], poor general health has increased significantly in all age groups, education groups, and regions. Women increased significantly more than men and rural regions increased more than urban regions. From 2000, women with poor general health have increased 2.6% and, from 2008, the difference between women and men has increased. In comparison, Martin et al. [26] showed less decline in poor health at younger ages and widening health disparities by income. Abebe et al. [27] concluded that the economic crisis of 2005–2011 accounted for an increasing trend in fair and poor self-rated health among the general working-age population of Europe. The present study’s results of trends in poor general health in all groups, in particular among women, are important, not least due to the association between poor general health and mortality, which needs to be addressed in societal efforts.

### 4.2. Overall and Work-Related Stress

Not expected and contradicting other studies, experiences of work-related stress in this study decreased between 2000 and 2016 [2,3,4,5,6]. Work-related stress has decreased significantly in all groups, but women have decreased significantly less than men and urban regions less than other regions. Overall stress (i.e., both work stress and private life stress) shows a somewhat different pattern, with few changes over time. These contradicting trends in work-related stress and overall stress put focus on the importance of taking a broader perspective of stress and carefully surveying whether the stress results from either the private role, such as parental responsibility or the work role, such as imbalance between job demands and control, or both, in order to design effective preventive efforts. The healthy worker effect possibly contributes to this pattern as the data are based on a working population. Moreover, the number of individuals on sick leave due to stress related ill-health increasing markedly the last decade [2,3,4,5,6] is not in line with the changes in self-reported stress in the present study, indicating that trends in sick leave behavior is worth further investigation. Indeed, self-reported stress reaction increasing in particular among the young [8,9] is supported in the present result of the young increasing modestly in self-reported overall stress. Another picture of trends in self-reported work stress is shown if the years between 2000 were considered as these years were characterized by large work-related changes, such as reorganizations and downsizing, in particular within the public sector [6,7]. This is indicated in the present study results when the years between 1995 and 2000 were included.

### 4.3. Loneliness

The stressor loneliness has increased significantly over the years with a prevalence rate increase similar to other studies [11,14]. The increase is particularly evident in women, the young, and people living in All other regions (than urban or rural), and more in women compared to men. Since 2003, younger individuals reported more loneliness than older individuals and, in line with previous literature [13], the prevalence of loneliness in older individuals was stable. Social media and solely living are potential explanations for the increase in loneliness in young individuals [13] and, considering that loneliness is a strong predictor of long-term morbidity and mortality in the general population [14], the increase in feelings of loneliness in young is serious and important to combat. 

### 4.4. Sleeping Problems

Sleeping problems are widely believed to impair health through their various effects on bodily systems, e.g., neuroendocrine, immune, and metabolic systems [16]. The present study results show that sleeping problems have increased significantly with a relative increase of almost 90% in all groups between 2000 and 2016 and significantly more in young and individuals with low education compared to older and high education groups. Similarly, a Norwegian study [18] showed a twofold increase in insomnia symptoms and tiredness from the 1990s to the end of the 2000s and a study in USA found insomnia symptoms in particular in women and individuals over 40 years increasing [20]. Another Swedish longitudinal study [19] also showed a twofold increase in sleeping problems from 1968 to 2004. That certain groups are suffering from sleep disturbances needs special focus and efforts. 

### 4.5. Combined Effect of Symptoms

The risk of having more than three symptoms has increased significantly from 2000 to 2016, in particular in individuals with low education and young individuals between 18 and 34 years. The proportion of women with more than three risk symptoms has increased significantly since 2010. Moreover, men were more likely to have no symptoms and less likely to have three symptoms, compared to women, both in the first year and in the last year of the study period. This is in line with the literature showing women and those belonging to a low socioeconomic group are at the highest risk for ill-health [6,7,26,27,28].

Limitations in the study are that the study was based on a selective sample of working adults, which may reduce the generalizability to other groups concerning the health-related factors in the study. However, the prevalence in the health factors are in parallel with other populations [18]. Other limitations are that the measures of health were self-reported and not psychometrically investigated. Being self-reported, there may be individual differences in how the measures, such as poor sleep, were interpreted. However, the measures are scientifically published [21,22] and evaluated in clinical settings through occupational health care with several hundred thousand performed tests since 1976. Strengths in the study are the large population of working adults and the long time period of 16 years, providing unique possibilities to analyze trends in demographic subgroups of age, gender, regions, and socioeconomic groups. 

## 5. Conclusions

To conclude, on the population level, sleeping problems and poor general health increased markedly while experiences of work-related stress decreased during 2000–2016. Overall stress and level of loneliness were unchanged at the population level. The risk of having more than three symptoms increased significantly from 2000 to 2016, in particular in the low socioeconomic group and young individuals, and the number of risk symptoms are higher in women compared to men. The hypotheses are partly confirmed, showing a negative trend in all health factors except in stress and loneliness at the population level. In the present sample of working adults, individuals with lower education, women, and young individuals experienced greater reductions in subjective health ratings than other education groups, men, and older individuals. That certain groups are suffering particularly from poor general health, stress, and sleep disturbances needs special focus and efforts. Moreover, the impact of social media and solitary living for feelings of loneliness, particularly for young people, warrants further research and efforts.

## Figures and Tables

**Figure 1:**
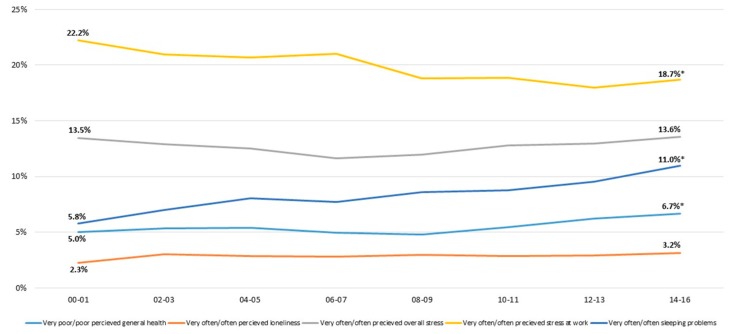
Change in standardized proportions of the five dichotomized outcomes between 2000–2001 and 2014–2016 in the total study population. * Significant trend between 2000–2001 and 2014–2016, *p* < 0.05.

**Table 1:** Distribution of sex, age, and length of education in the study population, 2000–2016.

		Sex		Age			Years of Education		
Year	N	Women	Men	18–34 y	35–49 y	50–74 y	≤9 y	10–12 y	>12 y
2000–2001	15,731	49%	51%	26%	40%	34%	13%	67%	20%
2002–2003	27,387	53%	47%	27%	40%	33%	12%	68%	20%
2004–2005	45,981	52%	48%	24%	42%	35%	11%	64%	25%
2006–2007	46,415	48%	52%	24%	42%	34%	11%	65%	24%
2008–2009	50,697	47%	53%	25%	41%	34%	10%	64%	25%
2010–2011	41,870	46%	54%	25%	44%	31%	10%	63%	27%
2012–2013	47,926	42%	58%	25%	44%	31%	9%	62%	30%
2014–2016	59,618	41%	59%	27%	41%	31%	8%	62%	30%
Totalt	335,625	46%	54%	25%	42%	33%	10%	64%	26%

During the period 2000–2016 (Figure 1), the results on the whole population show that poor general health increased significantly from 5% to 6.7% and sleeping problems from 5.8% to 11%. During the same period, work-related stress decreased from 22.2% to 18.7% (*p* < 0.05) while overall stress as well as loneliness remained unchanged.

**Table 2:** Change in OR (95% CI) per year for having ≥3 symptoms: any of poor or very poor general health, often or very often perceived overall stress, loneliness, or sleeping problems (2000–2016).

	Three or More Symptoms
	β (95% CI)
Total population	1.034 (1.027 to 1.040)
per year by sex	
Women	1.037 (1.029 to 1.045)
Men	1.029 (1.019 to 1.039)
per year by age-group	
18–34 years	1.052 (1.038 to 1.065)
35–49 years	1.030 (1.020 to 1.039) a
50–74 years	1.029 (1.018 to 1.041) a
per year by educational level	
≤9 years	1.056 (1.037 to 1.076)
10–12 years	1.032 (1.024 to 1.040) b
≥12 years	1.030 (1.017 to 1.043) b
per year by region	
Urban counties	1.030 (1.021 to 1.039)
All other counties	1.044 (1.033 to 1.056)
Rural counties	1.029 (1.015 to 1.043)
Adjusted for sex, age and educational level	

a β-value significantly different from 18–34 years. b β-value significantly different from ≤9 years.

## Supplementary Materials

The following are available online at https://www.mdpi.com/1660-4601/17/2/511/s1, Table S1. Prevalence in health related factors during the years 1995–1999, Table S2. OR (95%) for having no risk symptoms (left) and three or more symptoms (right) in the first four and last four of the study period in relation to sex, age-group, length of education and region.
