# Supplementary figures and images for: Self-Reported General Health, Overall and Work-Related Stress, Loneliness, and Sleeping Problems in 335,625 Swedish Adults from 2000 to 2016
Source: Int J Environ Res Public Health. 2020 Jan 14;17(2):511. doi: 10.3390/ijerph17020511 (PMC7014157; doi:10.3390/ijerph17020511)

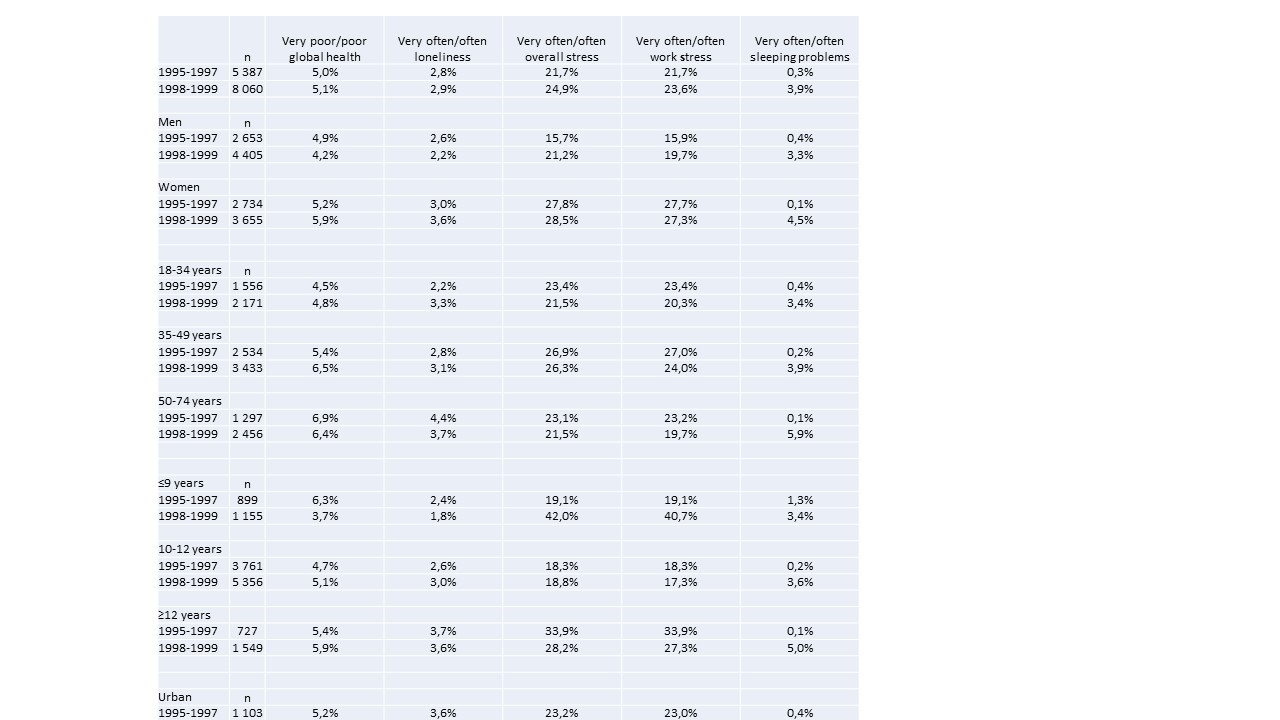

Supplement: Supplementary file 1 [file ijerph-17-00511-s001.zip › Supplement Table 1.JPG]

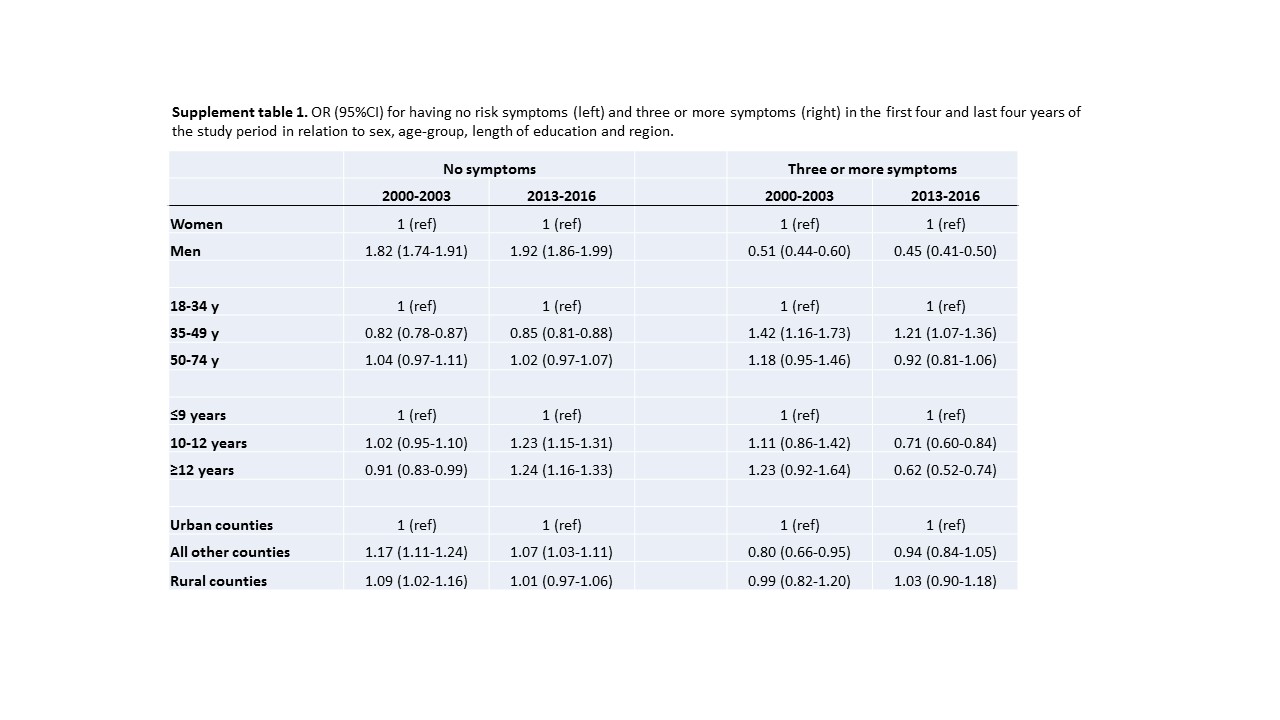

Supplement: Supplementary file 1 [file ijerph-17-00511-s001.zip › Supplement Table 2.JPG]
